# One Step Ahead—Attention Control Capabilities at Baseline Are Associated With the Effectiveness of the Attention Training Technique

**DOI:** 10.3389/fpsyg.2020.00401

**Published:** 2020-03-31

**Authors:** Ivo Heitland, Vincent Barth, Lotta Winter, Niklas Jahn, Alev Burak, Christopher Sinke, Tillmann H. C. Krüger, Kai G. Kahl

**Affiliations:** ^1^Department of Psychiatry, Social Psychiatry and Psychotherapy, Hannover Medical School, Hanover, Germany; ^2^Division of Clinical Psychology and Sexual Medicine, Hannover Medical School, Hanover, Germany

**Keywords:** metacognitive therapy, Attentional Training Technique, dose-dependent effects, Attentional Control Scale (ACS), MCT, ATT, attentional performance

## Abstract

**Background:**

Attentional control has been observed to play an important role in affective disorders by impacting information processing, the ability to exert top–down control in response to distracting stimuli, and by affecting emotional regulation. Prior studies demonstrated an association between attentional control and response to psychotherapy, thereby identifying attentional control as an interesting prognostic pre-treatment factor. Improving attentional control and flexibility is a cornerstone in metacognitive therapy (MCT), which is trained by the use of the Attentional Training Technique (ATT). However, as of yet, it remains unclear if pre-treatment attentional control is related to the effect of ATT.

**Methods:**

An aggregated sample of 139 healthy participants [study 1: 85 participants, mean age 23.7 years, previously published (Barth et al., 2019); study 2: 54 participants, mean age 33.7 years, not previously published] performed an attentional performance test battery before and after applying ATT. Before ATT was administered, attentional control was measured using a well-established self-report instrument, i.e., the Attentional Control Scale (ACS; Derryberry and Reed, 2002). ATT was given in 2, 4, or 15 doses and compared to sham ATT. The test battery comprised a selection of established neurocognitive tasks: emotional dot probe, Stroop, 2-back, and dichotic listening.

**Results:**

Sham ATT showed no interaction with ACS score on performance outcome in all tests. At four doses of ATT, ACS score was associated with training response, i.e., subjects with high self-reported attentional control before training showed the largest improvements post-training (all *P*-values <0.05; see Figure 3). At 2 and 15 doses of ATT, the ACS score was unrelated to training response.

**Conclusion:**

This is a first attempt in understanding the optimal dosage in which ATT should be administered dependent on the individual characteristics of each subject pre-training. The current data suggest self-reported attentional control pre-training as a marker to determine an optimal individual ATT training profile. Future studies should investigate if other domains of metacognitions also interact with training outcome and evaluate the extent to which this relationship transfers to clinical samples. If successful, assessing attentional control prior to treatment in clinical samples could be of use regarding personalized therapy plans and treatment outcome.

## Introduction

Attentional control (AC) is described as the general capacity to control attention in relation to positive or negative information (Derryberry and Reed, 2002). AC comprises focusing and shifting attention. Derryberry and Reed (2002) describe attentional shifting, also referred to as orientation, as a process of attentional disengagement from one target, moving attentional resources to a new target and subsequently engaging the new attentional target. Attentional focusing is to the ability to intentionally hold attention to desired stimuli and to avoid shifting attention to irrelevant or distracting stimuli (Derryberry and Rothbart, 1988).

Several studies demonstrated that anxious participants with good AC were better in disengaging from threatening information (Fox et al., 2001, 2002; Derryberry and Reed, 2002). Furthermore, others did report that attentional focusing can predict anxiety scores in healthy participants, while attention shifting abilities can predict depression scores in the healthy population (Ólafsson et al., 2011). Accordingly, AC allows anxious persons to limit the impact of threatening information, whereas those with poor AC are more likely to be preoccupied by threatening cues (Derryberry and Reed, 2002; Mathews and MacLeod, 2005). In contrast, participants with higher trait anxiety and worrisome thoughts take longer to switch attention from neutral information to emotional (Johnson, 2009). Of note, the relationship between anxiety and AC seems to be bi-directional. That means that not only does high AC function as a buffer for anxious pathologies, but also, anxiety itself can decrease AC by impairing efficient functioning of the goal-directed attentional system (Eysenck et al., 2007). Following that line of thought, Eysenck et al. (2007) stated that potential adverse effects of anxiety depend on AC involving the inhibition and shifting of attention. These processes of initially shifting attention toward threat cues and subsequently holding attention toward the threat is explained by a dual process view (Mathews and MacLeod, 2005). Bottom–up activation of threat representations within a salience network could explain the initial attention shift toward emotional cues (Öhman and Mineka, 2001). Attention to threat cues in anxiety is explained by top–down activation of competing representations related to other goals by an AC system (Matthews and Mackintosh, 1998).

In addition to findings regarding anxious traits, AC seems to play an important role in a number of affective disorders like anxiety and depression (Gotlib et al., 2004; Eysenck et al., 2007; Buckman et al., 2019). Poor AC is associated with impaired emotion regulation in depression (Joormann and D’Avanzato, 2010; Koster et al., 2011; Joormann and Michael Vanderlind, 2014; DeJong et al., 2019). Similar to anxiety, impaired attentional disengagement from negative self-referent information is linked to depressive symptoms like rumination (Koster et al., 2011). Buckman et al. (2019) showed that self-reported AC pre-treatment does predict the level of depressive symptoms post-treatment as well as the risk of relapse to depression. Koster et al. (2011), continuing that line of thought, suggest improving AC first in order to change one’s habitual style of thinking in depression, while only verbal interventions might not aim directly at impaired AC. In conclusion, this suggests AC as an interesting prognostic pre-treatment factor regarding anxious and depressive pathologies.

One model describing the connection between affective disorders and (impaired) AC is the Self-Regulatory Executive Function model (S-REF; Wells and Matthews, 1994). The S-REF comprises three interacting levels: a level of automatic and reflexively driven processing units, a level of attentional demanding and voluntary processing, and a level of stored knowledge or self-beliefs (Wells and Matthews, 1994). Self-regulation is processed in a limited capacity at the voluntary processing level and relies on voluntary attention for execution (Wells and Matthews, 1994). Operations processed by the controlled processing system are guided by self-knowledge or self-beliefs (Wells and Matthews, 1994). In the S-REF model, attentional biases are a consequence of threat monitoring strategies in anxiety maintained by dysfunctional metacognitive beliefs. In patients that focus on channels associated with threat, demanding resources of voluntary attention can lead to impaired AC. AC strategies, and behind these, dysfunctional beliefs, might be a stress coping strategy. Furthermore, the style of thinking and coping is able to cause prolonged maladaptive emotional responses (Wells and Matthews, 1996).

Improving AC and flexibility is a cornerstone in metacognitive therapy (MCT), which is trained by the use of the Attentional Training Technique (ATT; Wells, 1990, 2007). The ATT is based on the S-REF model and aims to improve attentional flexibility by training selective attention, attentional switching, and divided attention. The ATT has been proven as an efficient standalone treatment for depression and anxiety (see Knowles et al., 2016). In a previous study (Barth et al., 2019), we demonstrated that two and four doses of ATT improve attention performance regarding auditory information (dichotic listening task) and attentional disengagement (emotional dot probe) in comparison to an active control group. Of note, a recent study demonstrated that only a single ATT session could already improve AC measured by the Stroop task (Fernie et al., 2019).

Derryberry and Reed (2002) developed a self-report questionnaire to measure AC as the general ability to deliberately control, focus, and shift attention. This Attentional Control Scale (ACS) is used in this study to investigate the potential predictive power of pre-treatment attentional abilities on performance outcomes. Note that in the theoretical framework of the S-REF model, a self-report measurement such as the ACS potentially not only measures the self-evaluation of one’s own attention abilities but also may measure metacognitive beliefs of participants about their ability to control, focus, and shift attention.

This study aims to investigate if differences in pre-treatment attentional capabilities will affect outcome differences depending on different doses of ATT. Therefore attentional and executive functioning in healthy controls was tested before and after different doses of attentional training. We hypothesize that the better the self-rated attention control, the higher the improvement through attentional training.

## Materials and Methods

All study procedures were approved by the local ethical committee of Hannover Medical School. Written informed consent in accordance with the Declaration of Helsinki was provided by all subjects. All subjects received monetary compensation for participation. The current study comprises an aggregated sample derived from two independent studies performed in our lab, i.e., study 1 (Barth et al., 2019) and study 2 (Jahn et al., 2020). In total, the aggregated sample consists of 139 healthy participants.

### Procedure

**FIGURE 1:**
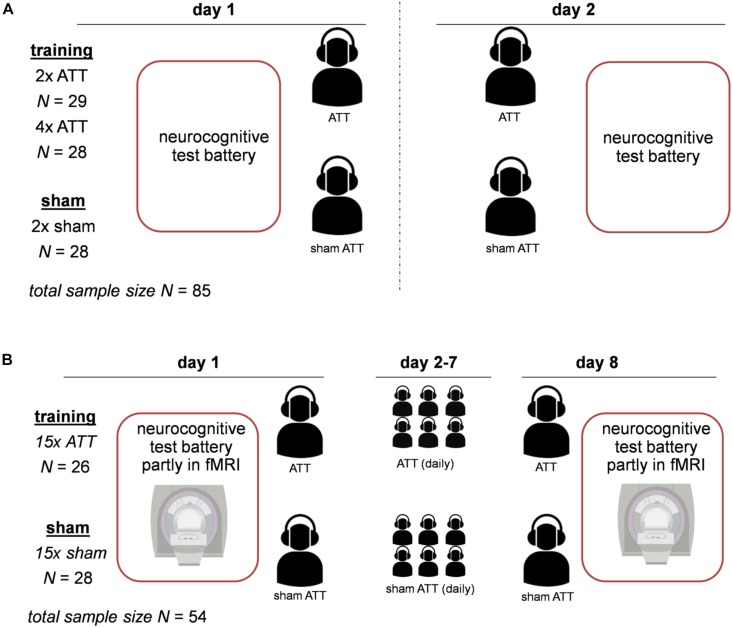
Experimental design for both studies. Panel **(A)** displays the design of study 1; Attentional Training Technique (ATT)/sham ATT was performed on two consecutive days. Panel **(B)** shows the design of study 2; ATT/sham ATT was performed on day 1 and day 8 in the lab, and in between (day 2–day 7), subjects were trained with ATT/sham ATT at home.

Both studies were designed as randomized placebo-controlled trials. The procedures for both studies were largely similar. For an overview of the design of both studies, see Figure 1. Before the experiments started, participants were reported to be free of psychiatric diagnoses according to International Statistical Classification of Diseases and Related Health Problems (ICD-10) criteria in the last 3 months. In study 1, this was assessed using a short clinical interview with a clinician. In study 2, the German version of the Structured Clinical Interview for Diagnostic and Statistical Manual of Mental Disorders (DSM-IV) (SCID) screening was used. In both studies, subjects first filled in the ACS questionnaire and performed a neurocognitive test battery at baseline on a computer. Participants were then subjected to either ATT or sham ATT in the lab. Subjects were trained with ATT/sham ATT on two consecutive days (study one) or on eight consecutive days (study 2). On the last day after the ATT training session, the neurocognitive test battery was performed again.

### Sample 1

The first sample consists of 85 healthy students recruited from a German university (for details, see Barth et al., 2019). Participants were between 18 and 37 years of age (mean age: 23.7, *SD* = 3.6). Data of four subjects were discarded due to incomplete or invalid recordings. For a detailed description of all experimental procedures for the first sample, see Barth et al. (2019) and Figure 1. The experiment took place on two consecutive days (see Figure 1). In this sample, the ATT/sham ATT manipulation comprised groups of two doses of ATT, four doses of ATT, and sham ATT with two doses of sham training. The four-dose ATT group started the training on the first day with two sessions of training after finishing the test battery. The two-dose ATT and sham ATT groups only performed the test battery on day 1. On day 2, all groups started with two sessions of training or sham training and completed the task battery afterward (see Figure 1).

### Sample 2

The second sample consisted of 54 healthy participants ranging from 25 to 50 years of age (mean age: 33.7, *SD* = 7.7). Of the subjects, 64.8% were female; 35.2% were male. Data of four subjects were discarded due to an incidental white matter lesion finding on MRI (*N* = 1 in the sham ATT group), depressive symptoms in the SCID screening at baseline (*N* = 1 in the sham ATT group), misunderstanding of task instructions (*N* = 1 in the sham ATT group), and falling asleep during functional magnetic resonance imaging (fMRI) measurement (*N* = 1 in the ATT group). In comparison with study 1, the main objective in this sample was to evaluate the neurobiological effects of ATT; therefore, a part of the neurocognitive test battery was conducted in an fMRI scanner. The fMRI data are currently being processed and will be presented in a separate report (Jahn et al., 2020). The first training and the last ATT training were performed in the lab, comprised two doses of ATT each, and were done 8 days apart (see Figure 1). In between, subjects were instructed to perform two doses of ATT daily at home (see Figure 1). Participants provided written documentation of these ATT trainings at home. The average amount of completed trainings was *M* = 14.8 *SD* = 2.1 for the sham ATT group and *M* = 14.9, *SD* = 2.2 for the ATT group.

### ATT and Sham ATT

The ATT was presented using a standardized German audio file as described in the MCT manual (Wells, 2009). The ATT’s main focus is to improve AC and attentional flexibility (Wells, 2009). The ATT comprises three auditory attentional exercises: selective attention, attention switching, and divided attention. Each training session in lab and at home consisted of hearing the ATT audio file twice, in which only audio file 1 contained explanations of the upcoming training (for detailed description, see Barth et al., 2019). One session of ATT lasts 12 min in total, with instructions (1 min), selective attention exercise (5 min), rapid attention switching (5 min), and divided attention (1 min). The sham training group listened to a non-treatment audio file, which comprised the same sounds, duration, and intensity as in the ATT but without verbal instructions. In this report, four groups were investigated in total, i.e., 2, 4, and 15 doses of ATT and sham ATT (2 and 15 doses combined).

### Attentional Control Scale (ACS)

The ACS (Derryberry and Reed, 2002) is a self-report measure of AC, attentional focusing, and attentional shifting. It consists of 20 items rated on a four-point Likert scale (almost never, sometimes, often, always). The questionnaire was developed as an instrument to measure the general capacity for AC, with high sum scores indicating good AC. The ACS comprises two subscales measuring the capability to focus attention (ACS focus) and to shift attention dynamically (ACS shifting). The ACS questionnaire was completed on the first day before the test battery was performed.

### Neurocognitive Test Battery

The neurocognitive test battery comprised a number of well-validated tasks to assess attentional performance. In both samples, these were a dichotic listening task, an emotional dot probe task, a Stroop task, and a 2-back task. Additionally, a 3-back task and the attentional network task were included in sample 1. In sample 2, these tasks were excluded to account for the longer duration of the experimental procedures due to the fMRI measurement, and as the data from study 1 did not warrant further use. All tasks started with written instructions and a short exercise block to ensure participants followed the instructions.

### Dichotic Listening

The dichotic listening task was used as described in Asbjørnsen and Hugdahl (1995). The task was used to test whether ATT improved selective attentional focusing in the domain of auditory processing. Participants had to focus on one ear (first trial, left ear; second trial, right ear) while listening to different consonant–vowel syllables. These were presented simultaneously on both ears via headphones. For a detailed description of the task, see Barth et al. (2019). As described there, the outcome variable was the weighted mean of all left and right ear correct reaction times in milliseconds in the forced listening condition. The T2 minus T1 difference of these weighted means was subject to analyses. Due to incomplete or invalid recordings, group sizes in the analyses were: sham ATT, *n* = 51; 2 doses of ATT, *n* = 27; 4 doses of ATT, *n* = 27; and 15 doses, *n* = 25.

### Emotional Dot Probe

The emotional dot probe was utilized to measure selective AC in the visual domain. For detailed description of the task procedure and details, see Barth et al. (2019). The test procedure was similar to Donaldson et al. (2007). A word pair, with one above a central fixation point and one below, was displayed for 1,000 ms. In study 2, the word pairs and targets were presented left and right of the fixation cross in order to better match the used response buttons located at the left and right index finger. Due to a prolonged inter-stimulus-interval (ISI) for fMRI analyses, only 90 trials were presented (45 congruent and 45 incongruent) in the fMRI version of this task. For both versions, in each trial, one word had a negative valence, and the other was neutral. After the words disappeared, participants had to react to a target (asterisk), which appeared either in the position of the emotional word or in the position of the neutral word for 2 s. Fifty trials were presented per condition. As there is a bias in humans to allocate attentional resources toward salient and emotional stimuli (Macleod et al., 1986), the condition in which the asterisk appears at the location of the emotional word is typically referred to as congruent, as attention is already allocated at the target location. In contrast, the condition in which the asterisk appears in the location of the neutral word is typically referred to as incongruent and requires attentional disengagement, as attention is allocated at the opposite location, leading to longer reaction times compared to the congruent condition. In study 1, subjects completed the emotional dot probe while sitting in front of a computer. In study 2, this task was conducted while participants were lying in the MRI scanner. Subjects had to press two buttons with a computer mouse (study 1) or two input devices for each hand with two buttons on each (study 2). The stimuli were presented on a 32-inch display from Neuro-Nordic-Lab (NNL) at the end of the scanner; participants were able to see the screen through a mirror right above their head. Outcome variables were the mean reaction times in milliseconds. As an index of task improvement, the T2 minus T1 difference for the reaction times was analyzed. Due to incomplete or invalid recordings, group sizes in the analyses were: sham ATT, *n* = 47; 2 doses of ATT, *n* = 23; 4 doses of ATT, *n* = 24; and 15 doses, *n* = 25.

### Stroop Task

The Stroop task (Stroop, 1935) was used to measure selective attention and executive control as inhibition described in the parallel distribution processing model (see MacLeod, 1991). Stroop task presented capitalized color words (RED, YELLOW, GREEN, and BLUE) against a black background. Two conditions were conducted: in congruent trials, words were presented in their matching color (e.g., the word BLUE in blue). In incongruent trials, words were presented in a mismatching hue of the other three colors (e.g., BLUE in red). Participants had to indicate the hue of the words and ignore the semantic meaning of the color words. One hundred trials were presented, which were equally distributed across conditions (50 congruent and 50 incongruent trials). For a detailed description, see Barth et al. (2019). In study 1, the Stroop task was performed in the lab while subjects sat in front of a computer. In study 2, the Stroop task was conducted while participants were lying in the MRI scanner. Participants had to press two buttons with the thumb and index finger of their left hand (red and yellow) and two buttons with the thumb and index finger of their right hand (blue and green). To ensure full understanding of the task, color–button correspondences were displayed at both sides of the screen on paper. The primary outcome variable was the mean reaction times of congruent hits and mean reaction times of incongruent hits in milliseconds. As an index of Stroop task improvements, the corresponding T2 minus T1 differences were analyzed. Due to incomplete or invalid recordings, group sizes in the analyses were: sham ATT, *n* = 50; 2 doses of ATT, *n* = 27; 4 doses of ATT, *n* = 27; and 15 doses, *n* = 25.

### 2-Back

The N-back task measures working memory (WM) performance as described in Braver et al. (1997). We used a sequential letter task in this version of 2-back, in which participants had to determine if the current letter was identical to the letter two trials before (see Braver et al., 1997, p. 57, for detailed description). Each displayed letter was presented for 1,500 ms, followed by a 500 ms pause before the next letter appeared. Participants had to respond to every letter and identify if the current letter was a target (identical with the letter two trials before) or a non-target by pressing two keyboard buttons. All 26 alphabetical letters were used in a randomized order, with no more than two targets in a row (for detailed description, see Barth et al., 2019). In total, 150 letters were presented, with 50 targets and 100 non-targets. Outcome variables were the means of hits of target reaction times in milliseconds. The T2 minus T1 difference of these means was subject to analyses. Due to incomplete or invalid recordings, group sizes in the analyses were sham ATT, *n* = 49; 2 doses of ATT, *n* = 25; 4 doses of ATT, *n* = 27; and 15 doses, *n* = 25.

### Statistical Analyses

All statistical analyses were conducted with SPSS Statistics version 23.0 (IBM Corp., Armonk, NY, United States). An alpha of 0.05 was used. To investigate if ACS score at baseline modulated ATT-dependent performance improvements, non-parametric correlations between ACS score and performance improvements (T2 - T1) were computed per task (dichotic listening, emotional dot probe, Stroop, and 2-back) and dosage (sham, 2 × ATT, 4 × ATT, 15 × ATT). Of note, the outcome (significant vs. non-significant) of all correlational analyses presented in the following did not depend on the choice of parametric (Pearson’s r) vs. non-parametric correlations (Spearman’s rho). That means all correlations reported in the following that were significant for Spearman’s rho were significant when analyzed using Pearson’s r. Furthermore, all non-significant results with regard to Spearman’s rho remained non-significant when Pearson’s r was computed.

## Results

### Sample Characteristics

There were no differences in the ACS total score at baseline between all ATT groups (*p* = 0.23; sham ATT: *M* = 59.0, *SD* = 7.32; 2 doses of ATT: *M* = 56.22, *SD* = 6.25; 4 doses of ATT: *M* = 56.74, *SD* = 8.17; and 15 doses: *M* = 60.08, *SD* = 7.02). In addition, sex was evenly distributed across all groups [*x*^2^(4) = 0.895, *p* = 0.93]. As expected from the different inclusion criteria per study, the sample used for study 2 was significantly older than in study 1 (*p* < 0.01).

### Manipulation Check: Sham-Controlled ATT Effects Across Samples

As reported earlier (Barth et al., 2019), improvements across tasks were larger for the experimental groups that performed ATT than for the sham ATT groups. A brief overview of these results is presented here; for a more detailed description, please see (Barth et al., 2019).

In sample 1, participants who received two doses of ATT and four doses of ATT showed larger improvements (T2 - T1) in the dichotic listening task [*F*(1,75) = 5.17, *p* = 0.026, $\eta_{\text{p}}^{2}$ = 0.065], in the emotional dot probe task [only four doses: *F*(1,42) = 4.97, *p* = 0.031, $\eta_{\text{p}}^{2}$ = 0.106], and, as a trend, in the Stroop task [*F*(1,75) = 3.12, *p* = 0.081, $\eta_{\text{p}}^{2}$ = 0.040] when compared to sham ATT. There were no significant ATT vs. sham ATT effects with regard to the 2-back task (*p* = 0.77).

**FIGURE 2:**
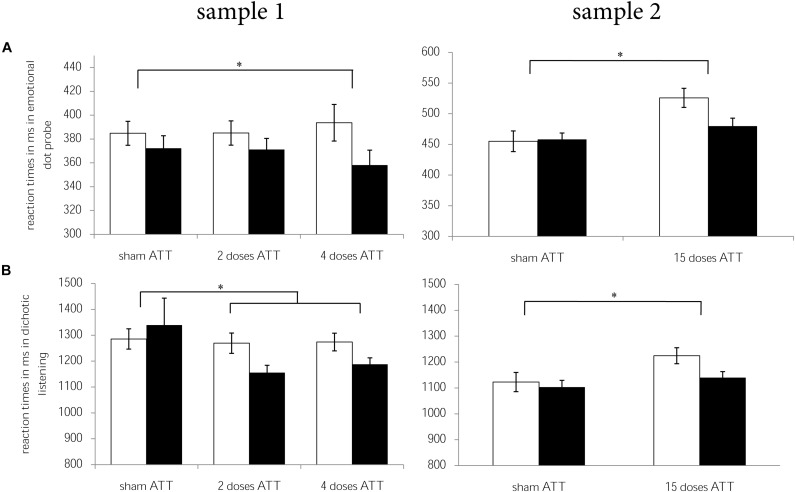
Reaction times for T1 (white) and T2 (black) per ATT group (sham ATT, 2 doses of ATT, 4 doses of ATT, 15 doses of ATT) are displayed for **(A)** dichotic listening and **(B)** emotional dot probe per sample (sample 1 = left, sample 2 = right). Error bars indicate ± 1 standard error of the mean (SEM). *p < 0.05.

Detailed analyses of ATT vs. sham ATT data including fMRI will be presented in another report (Jahn et al., 2020). In brief, we replicated the ATT vs. sham ATT effects reported in sample 1. That means that subjects who received ATT showed larger improvements (T2 − T1) with regard to dichotic listening [*F*(1,45) = 4.158, *p* = 0.047, $\eta_{\text{p}}^{2}$ = 0.085] and the emotional dot probe task [attentional disengagement: *F*(1,44) = 8.265, *p* = 0.006, $\eta_{\text{p}}^{2}$ = 0.158] than the sham-control group. There were no effects with regard to the Stroop task (*p*’s > 0.102) and the 2-back task (*p* = 0.457). An overview of the ATT vs. sham ATT effects from both samples is presented in Figure 2.

**FIGURE 3:**
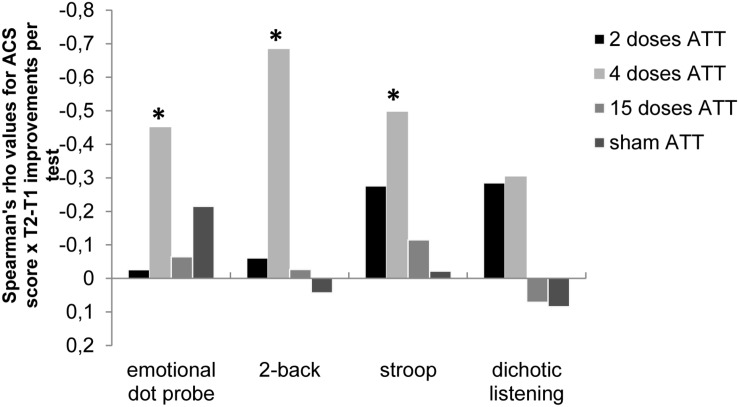
Spearman’s rho correlation values (*r*) of Attentional Control Scale (ACS; Derryberry and Reed, 2002) scores × performance improvements (T2 – T1) for the respective neurocognitive tests; i.e., the emotional dot probe task, the 2-back task, the emotional Stroop task, and the dichotic listening task are shown dependent on ATT dosage (2, 4, or 15 doses). This illustrates the dose-dependent relationship between self-reported attentional capabilities at baseline and dose-dependent neurocognitive performance improvements, with a significant association of ACS only at four doses of ATT. *p < 0.05.

### ACS as a Factor in ATT-Dependent Performance Improvements per Dose

In subjects who performed sham ATT, there were no associations between ACS score at baseline and neurocognitive performance improvements (all *p*-values > 0.147). Furthermore, there were no associations between ACS score at baseline and neurocognitive performance improvements in the 2 × ATT group (all *p*-values > 0.149) or the 15 × ATT group (*p* > 0.421).

In subjects who performed ATT four times, however, a high ACS score at baseline was associated with larger performance improvements in the emotional dot probe task [*r*_*s*_(24) = −0.451, *p* = 0.027], the Stroop task [*r*_*s*_(27) = −0.479, *p* = 0.009], and the 2-back task [*r*_*s*_(27) = −0.684, *p* < 0.001]. ACS total score was not associated with improvements of dichotic listening reaction times in the four-dose ATT group. An overview of these results is displayed in Figure 3.

## Discussion

The present study investigated if and to what extent individual differences in self-reported AC at baseline are associated with neurocognitive performance improvements after having performed ATT or sham ATT. For that purpose, two independent samples completed a baseline assessment of AC followed by a neurocognitive test battery and were then subjected to various doses of ATT (2, 4, 15, and a sham group). One day (sample 1) or 1 week (sample 2) later, they returned to the lab to complete the neurocognitive test battery again. In both samples, subjects showed larger improvements in the neurocognitive assessments after ATT than after sham ATT. Of note, this effect was unrelated to ATT dosage, meaning ATT-dependent improvements were not larger at 15 doses of ATT than at 4 doses of ATT. This might be attributable to a ceiling effect. As healthy subjects typically report higher AC than patients and do not suffer from a cognitive attentional syndrome (CAS), four doses of ATT might be all that’s needed in improving attentional performance in that sample, with no additional benefits of more training. There were no ATT-dependent improvements in the 2-back task. As previously discussed in Barth et al. (2019), ATT does seem to train attention processes rather than basic WM performance, which is the process measured during the 2-back task. Hence, the absence of an ATT × 2-back improvement is consistent with that line of thought and previous findings (see Owen et al., 2005; Schmiedek et al., 2014).

Interestingly, the ATT-dependent improvement of neurocognitive performance was modulated by AC at baseline. Subjects who reported high AC pre-training showed larger neurocognitive performance improvements after only four doses of ATT, while no effects of pre-training AC were observed at 2 or 15 doses of ATT or after sham ATT. To our knowledge, this is the first reported link between pre-training AC and benefits of the ATT training, and underlines the importance of assessing pre-training individual differences in AC when ATT is applied.

Several mechanisms might be responsible for this effect. The ACS is a self-report instrument assessing AC capabilities, i.e., to focus and to switch attention. These are processes that ATT specifically aims to improve. As such, having a solid foundation of AC before being subjected to ATT might allow for an easier integration and application of ATT. Like with many other training programs, getting familiar with the program and getting used to the structure of the training is essential to integrate the learning experience. High pre-training levels of AC might allow for a faster switch from “getting used to” to training attentional flexibility. Therefore participants with high levels of AC might profit faster from training ATT. While ATT might be most beneficial for subjects with low baseline AC on the long-term, this group might simply need more training to achieve similar effects than an average- or high-AC group.

Of note, several different questionnaires have been studied to assess self-reported AC and metacognitive beliefs regarding attentional capabilities. The ACS stems from research on attentional biases and threat monitoring, which is most prominently found in anxiety disorders (Derryberry and Reed, 2002). Traditionally, the ACS is viewed as a measurement for AC capabilities rather than the corresponding (meta)cognitive beliefs. Recent studies (e.g., Quigley et al., 2017) have raised questions regarding that view by demonstrating a dissociation between the ACS and corresponding behavioral measurement for AC. Thereby, they made the suggestion that the ACS might be more closely related to perceptions and beliefs regarding AC than actual AC capabilities. This fits with the observation that the most consistent associations with the ACS have been reported regarding anxiety and depression (Ólafsson et al., 2011; Reinholdt-Dunne et al., 2013, 2019; Judah et al., 2014). In line with these findings, studies have shown that anxious and depressed individuals display negatively biased beliefs about themselves and their abilities, including AC (Beck et al., 1979; Chambless and Gillis, 1993; Spada et al., 2010; DeVito et al., 2019). Another questionnaire, the Meta-Cognition Questionnaire (MCQ; Cartwright-Hatton and Wells, 1997), was developed for a broader range of psychopathologies and aims to measure beliefs about worry, threat monitoring, and the controllability of thoughts. Future research has to clarify if AC as measured by questionnaires is more related to measureable attentional capabilities or, rather, one’s confidence and opinion regarding AC. If metacognitions are indeed measured with the ACS, our findings are in line with the concept that AC and flexibility are influenced by the metacognitive beliefs a person has. Those subjects who were more confident regarding their AC benefited sooner than those who had poorer beliefs about their AC.

Mechanistically, the strongest effects of pre-training AC-dependent ATT change were found in the 2-back task, even though no overall differences between the ATT and sham ATT groups were found. Attentional performance, i.e., focusing attention on relevant tasks while processing previous stimuli in the WM in the current case, seems to be associated with AC abilities at baseline. A similar effect was found in the emotional dot probe task. Participants with good AC at baseline were faster in focusing and reacting to targets with emotional valence after completing ATT, while there was no ATT effect on attentional disengagement. This might be due to larger voluntary attention resources in participants with good AC, which might allow them to benefit even more from training with four doses of ATT. This is in line with the theoretical underlying mechanisms of the S-REF model (Wells and Matthews, 1996) stating impaired AC as a consequence of demanding voluntary attention resources by inflexible attention and a heightened threat bias. Consistent with these statements, ATT-dependent improvements regarding attentional disengagement from irrelevant stimuli in the incongruent condition in the Stroop task were largest in high-ACS subjects. It seems that participants with good AC benefit more from training with four doses of ATT, which is shown in faster disengagement from irrelevant stimuli. As Fernie et al. (2019) stated, AC is not necessarily bound to emotional stimuli but rather more generally to disengaging from irrelevant stimuli. In the dichotic listening task, there was no modulation of ACS score at baseline on ATT-dependent improvements. The absence of an ACS effect in this task might be due to the modality overlap, meaning that training in the auditory modality as done in ATT and subsequently performing an auditory task might be significantly easier. Following that line of thought, ATT-based training effects might already be rather high regardless of poor AC.

With regard to dosage effects, we found no general advantage of 15 doses of ATT > 4 doses of ATT, as all room for improvement seemed to be covered by four doses of ATT already. Hence, the absence of an ACS modulation at 15 doses suggests a potential ceiling effect. Using that amount of training, pre-training differences might have evened out and no longer play a crucial role in ATT-based improvements. Typically, a variety of treatment effects follow an inverted u-shape dose response curve. This phenomenon was first described by Yerkes and Dodson (1908) regarding arousal and performance and has since been translated to, amongst others, behavioral pharmacology [see Calabrese (2008) for an overview], the neurobiology of human learning (Baldi and Bucherelli, 2005), and optimal patient–therapist relationships during psychotherapy (Dinger et al., 2009). In all these examples, the “sweet spot” for optimal treatment benefits lies in the middle of the distribution, with the medium intensity, duration, or dosage of treatment having the highest relative benefits. In the current study, a link for the optimal training benefits was already found at four doses of ATT, with no benefits of 11 additional doses using a healthy sample. Of note, there were no disadvantages in additional ATT sessions, as effects of 4 doses of ATT and 15 doses of ATT were comparable.

Hence, four doses of ATT was shown to be the optimal dosage for a healthy sample with relatively normal AC capabilities at baseline. In a clinical sample with potentially lower pre-treatment AC and greater problems regarding attentional flexibility, the optimal ATT dosage might be much higher. Following that line of thought, in a clinical setting, it might be worthwhile to account for baseline differences in AC when planning the dosage or when handling a patient’s expectations. This idea is in line with findings from a recent clinical study (Buckman et al., 2019). In a small cohort of depressed patients, baseline ACS predicted treatment response as well as residual depressive symptoms post-treatment and relapse rate, independent of symptom severity at the beginning. Moreover, clinical improvements were accompanied by an increase in ACS score from pre- to post-treatment, further underlining the importance of AC.

Certain limitations should be taken into account when interpreting the results of the current study. First, while the sample size of the aggregated samples used here is considerable (*N* = 135), larger follow-up studies are needed to fully elucidate the relationship between pre-training AC and ATT effects. Second, we only used four different ATT dosages, i.e., 2, 4, and 15 doses of ATT plus a sham-control group. While that approach allowed for finding a dose-dependent effect of ATT when pre-training AC was taken into account, a more elaborate design could allow for a more complete understanding and to potentially discover “sweet spots” for individual training profiles based on pre-training AC. This might be done using either a sham-controlled within-subject design or more ATT dosage groups (e.g., 1 dose of ATT to 10 doses of ATT). Third, the duration between pre- and post-assessment was either 1 day or 1 week. This does not allow for conclusions regarding longer as well as in-between time spans, which remains an interesting target for future studies. Fourth, both samples were significantly different in age, which limits the comparison of the 2- and 4-dose (sample 1) with the 15-dose (sample 2) group. Moreover, measurements for sample 2 took partly place in the MRI, leading to experimental changes and slightly different reaction times at baseline. This was accounted for by using reaction time improvements from T1 to T2 as an outcome measurement for all tasks in question. Fifth, the current study is limited to healthy participants. Translation of our findings to a clinical sample remains a very important task for the future. This also seems essential for using individual pre-treatment AC characteristics as potential biomarkers in determining individual ATT profiles in clinical practice. Sixth, note that this study combines data from two samples, with one previously published (Barth et al., 2019) and the other one being measured in the fMRI scanner. Due to the exploratory nature of this study, we did not correct for multiple comparisons, which would have slightly impacted the results for the pre-training AC × ATT findings. One out of three significant findings would narrowly exceed the alpha threshold (*P* = 0.027), while the other two survive Bonferroni correction (*P* = 0.009 and *P* < 0.001). As always advised regarding reports of novel associations, replication is preferred before stronger conclusions can be drawn.

For a long period of time, clinical practice has used a “one size fits all” mentality regarding various treatments and training techniques. In the last decades, numerous studies have demonstrated the importance of individual differences pre-treatment and their effects on treatment outcome (e.g., Haby et al., 2006; Lambert, 2017). This has led to a great spur in studies aimed at establishing biomarkers and usable heuristics for clinical practice, with great promise but, so far, limited success. We therefore believe that it is of utmost importance to continue the quest for personalized treatment plans in order to be able to offer optimal treatment guidelines and opportunities for patients. MCT and ATT in particular may be good targets for such an approach, as they are evidence-based and controllable psychotherapy methods with a clear definition.

Taken together, we here provide preliminary evidence suggesting pre-training AC as a factor in dose-dependent neurocognitive improvements following ATT. This suggests that self-reported AC pre-treatment might be used as a marker to determine an optimal individual ATT training profile. Future studies should replicate the current effects and investigate if other domains of metacognitions also interact with training outcome. Also, it remains crucial to evaluate the extent to which this relationship transfers to clinical samples. If successful, assessing AC prior to treatment in clinical samples could be of use regarding personalized therapy plans and evaluating treatment outcome.

## Data Availability Statement

The datasets generated for this study are available on request to the corresponding author.

## Ethics Statement

All study procedures were reviewed and approved by the local ethical committee of Hannover medical school. Written informed consent in accordance with the Declaration of Helsinki was provided by all subjects prior to participation.

## Author Contributions

CS, IH, and LW designed the experiments. NJ and AB recruited the subjects and collected the data under IH’s and VB’s supervision. VB, IH, NJ, and CS were responsible for data processing. VB, IH, and CS analyzed the data. All authors wrote the manuscript.

## Conflict of Interest

The authors declare that the research was conducted in the absence of any commercial or financial relationships that could be construed as a potential conflict of interest.
